# Diagnostic performance of stimulated plasma interferon-gamma inducible protein 10 (IP-10) for diagnosing latent tuberculosis infection in healthcare workers

**DOI:** 10.1038/s41598-026-51630-8

**Published:** 2026-05-04

**Authors:** Warangkana Keeratichananont, Theerapat Buppodom, Nawamin Pinpathomrat, Suriya Keeratichananont, Bunya Seeyankem, Ratchanon Sophonmanee, Sarayut L. Geater

**Affiliations:** 1 Division of Respiratory and Respiratory Critical Care Medicine, Department of Internal Medicine, Faculty of Medicine, Prince of Songkla University, Songkhla, 90110 Thailand; 2 Department of Internal Medicine, Faculty of Medicine, Prince of Songkla University, Songkhla, 90110 Thailand; 3 Department of Biomedical Sciences and Biomedical Engineering, Faculty of Medicine, Prince of Songkla University, Songkhla, 90110 Thailand; 4 NKC Institute of Gastroenterology and Hepatology, Faculty of Medicine, Prince of Songkla University, Songkhla, 90110 Thailand

**Keywords:** CXCL 10 protein, Human, Interferon-gamma release tests, Latent tuberculosis, Health personnel, Biomarkers, Diseases, Immunology, Medical research, Microbiology

## Abstract

In the absence of a definitive gold standard for diagnosing latent tuberculosis infection (LTBI) in healthcare workers (HCWs), alternative specific biomarkers are needed. This longitudinal cohort study with baseline testing evaluated the diagnostic performance of stimulated plasma interferon-γ inducible protein 10 (IP-10) for LTBI detection among HCWs in a high tuberculosis (TB)–burden setting, using interferon-γ release assays (IGRAs) as the reference standard. The study was conducted at Songklanagarind Hospital, Thailand, between 2021 and 2024, and included 64 healthy HCWs. Serum and stimulated plasma samples were collected, and IP-10 and IFN-γ concentrations were measured by ELISA. Nine HCWs (14.0%) were LTBI-positive. Stimulated plasma IP-10, but not serum IP-10, showed significant correlations with IFN-γ levels in TB1 (*r* = 0.719) and TB2 (*r* = 0.768; *p* < 0.0001) tubes. At cut-off values of ≥ 2,980.0 pg/mL for TB1 and ≥ 3,108.0 pg/mL for TB2, sensitivity was 100%, specificity was 96.0% and 94.5%, and overall accuracy was 96.8% and 95.3%, respectively. Based on either IGRA or stimulated plasma IP-10 cut-off levels, five of nine LTBI cases declined treatment; among these, one individual (20.0%) progressed to active pulmonary TB 16 months later. Stimulated plasma IP-10 shows high diagnostic performance for LTBI detection among HCWs and may serve as an alternative for LTBI screening in high–TB-incidence with universal BCG vaccination.

## Introduction

 Tuberculosis (TB) remains a major global health threat, causing 1.25 million deaths in 2023^1^. Approximately one-quarter of the world’s population is infected with *Mycobacterium tuberculosis* (MTB). Although most infected individuals are asymptomatic and classified as having latent TB infection (LTBI), 5–15% will progress to active TB during their lifetime, with the highest risk occurring within the first two years after infection. Consequently, identifying and treating LTBI is critical for TB control and a cornerstone of the WHO End TB Strategy^2–6^.

Among occupational healthcare workers (HCWs) with direct exposure to TB patients, a 2025 meta-analysis reported a pooled LTBI prevalence of 15.92%^7^. This prevalence increases to approximately 55% in low- and middle-income, high TB–burden countries, including Thailand^8–11^. HCWs with LTBI have up to a threefold higher risk of progressing to active TB compared to the general population. This increased risk may be primarily attributed to repeated occupational exposure to infectious TB patients, high bacillary load, aerosol-generating procedures, and suboptimal infection control measures, particularly in high TB-burden settings^9^. Accordingly, HCWs are recognized as a priority group in global TB control strategies. Under the WHO End TB Strategy, the goal is to reduce global tuberculosis incidence by 80% by 2030. Achieving this goal requires prevention, early detection, and prompt treatment of active TB, as well as identification and management of high-risk LTBI populations such as HCWs^2,6,9,12^. Furthermore, the CDC recommends LTBI testing and treatment for all HCWs unless contraindicated^12^.

Current LTBI diagnosis in HCWs relies mainly on the tuberculin skin test (TST) and interferon-γ release assays (IGRAs), which assess host immune responses to MTB antigens^3,9,13–17^. Although TST is simple and low cost, it requires trained personnel, multiple visits, and may yield false-positive results in BCG-vaccinated individuals or those exposed to nontuberculous mycobacteria (NTM)^13–15,18–20^. IGRAs are often preferred in these settings because they do not cross-react with BCG and are less affected by NTM exposure^21^. However, IGRAs have limitations, including reduced sensitivity in immunocompromised individuals, the need for specialized laboratory infrastructure, and higher costs^13–17^. As a result, IGRAs have not been widely implemented in low-income countries or included in Thailand’s Universal Coverage Scheme for LTBI screening^22,23^. Consequently, no gold-standard diagnostic test for LTBI currently exists, underscoring the need for novel biomarkers for LTBI diagnosis in HCWs^17^.

Interferon-γ–inducible protein 10 (IP-10; CXCL10), a CXC chemokine produced mainly by lymphocytes and monocytes in response to IFN-γ. It plays a key role in recruiting monocytes and Th1 cells to inflammatory sites following stimulation by MTB-specific antigens such as ESAT-6, CFP-10, and TB7.7. IP-10 can be measured in vitro in stimulated plasma or serum using ELISA-based assays, and individuals infected with MTB exhibit significantly higher IP-10 levels than unexposed controls. Notably, IP-10 is produced at substantially higher concentrations than IFN-γ^24,25^. A recent meta-analysis evaluating stimulated plasma IP-10 across diverse populations reported high diagnostic accuracy for LTBI, with a pooled sensitivity of 85%, specificity of 89%, and a positive likelihood ratio of 7.55 when TST or IGRAs were used as reference standards^26^. Additionally, IP-10 measurement is less costly and technically simpler than IGRAs, yields fewer false-positive results in BCG-vaccinated or NTM-exposed individuals, and shows lower interpretive variability than TST^23,27–29^. Consequently, IP-10 has emerged as a promising biomarker for distinguishing LTBI from uninfected individuals^29^.

To evaluate the diagnostic performance of IP-10 among HCWs, Rubbo et al. (2012) conducted a cross-sectional study involving 70 HCWs in France, a low–tuberculosis-incidence setting without universal BCG vaccination^30,31^. In their study, stimulated plasma IP-10 demonstrated strong discrimination between LTBI and uninfected participants.

Although IP-10 has shown promising diagnostic performance in various populations^29^, its utility in HCWs—especially in high-TB-burden settings—remains unclear. To address this, we evaluated IP-10 for detecting LTBI among adult HCWs in Thailand, a high-incidence TB country (with a TB incidence rate exceeding 100 cases per 100,000 population per year), characterized by rising NTM prevalence and universal BCG vaccination, using IGRAs as the reference standard^11,32,33^. The primary objective of this study was to evaluate the diagnostic performance of stimulated plasma IP-10 for LTBI detection in HCWs using IGRAs as the reference standard. The secondary objectives were to assess the correlations between stimulated plasma and non-stimulated serum IP-10 with IFN-γ levels.

## Results

Of 70 HCWs screened, 6 were excluded (3 declined, 1 had TB-compatible chest X-ray, 2 on steroids), leaving 64 participants. The demographic characteristics of the HCWs are presented in Table 1. Most were female (78.1%), median age 39 years, median work experience 5 years, and all had BCG vaccination. Nine HCWs (14.0%) were QFT-Plus positive (LTBI-positive in both TB1 and TB2 tubes), and 55 (86.0%) were negative (LTBI-negative); no indeterminate results occurred.

**Table 1:** Demographic characteristics of the 64 health care workers.

Characteristics of health care workers	Total participants (*N* = 64)
Female sex, n (%)	50 (78.1)
Age (year), median (IQR)	39 (30–44)
Hospital departments	
Inpatient department-medicine, n (%)	38 (59.4)
Outpatient department-medicine, n (%)	20 (31.3)
Emergency department, n (%)	6 (9.3)
Career groups	
Nurse, n (%)	47 (73.4)
Nurse aide, n (%)	9 (14.1)
Physician, n (%)	8 (12.5)
QuantiFERON-TB Gold Plus (QFT-Plus) assay results	
Positive, n (%)	9 (14.0)
Negative, n (%)	55 (86.0)
BCG vaccination in the first year of life	
Yes, n (%)	64 (100)

IQR, interquartile range; BCG, Bacillus Calmette–Guérin.

**Fig. 1:**
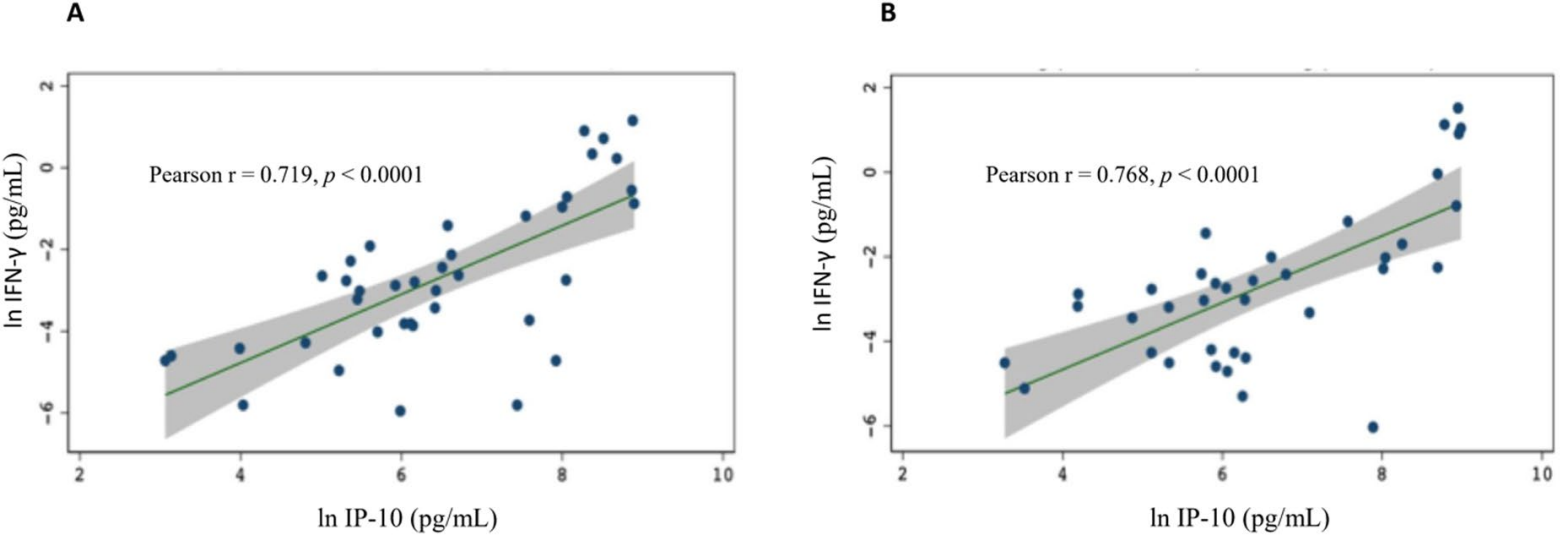
A scatter plots with superimposed linear regression lines illustrating the correction between stimulated plasma IFN-γ and stimulated plasma IP-10 concentrations in QFT-Plus TB1 (**A**) and TB2 (**B**) tubes. IFN-γ, interferon-gamma; IP-10, interferon-gamma inducible protein 10; QFT-Plus, QuantiFERON-TB Gold Plus.

Pearson’s correlation analysis demonstrated strong positive correlations between stimulated plasma IP-10 levels and IFN-γ responses in both TB1 (*r* = 0.719, *p* < 0.0001) and TB2 (*r* = 0.768, *p* < 0.0001) tubes (Fig. 1). HCWs with LTBI exhibited significantly higher median concentrations of TB antigen–stimulated plasma IP-10 compared with LTBI-negative HCWs (QFT-Plus TB1: 4,979.7 [3,163.4–5,871.6] vs. 352.3 [56.6–724.5] pg/mL; QFT-Plus TB2: 5,967.6 [3,251.1–7,541.1] vs. 370.0 [165.47–746.32] pg/mL; all *p* < 0.001).

**Fig. 2:**
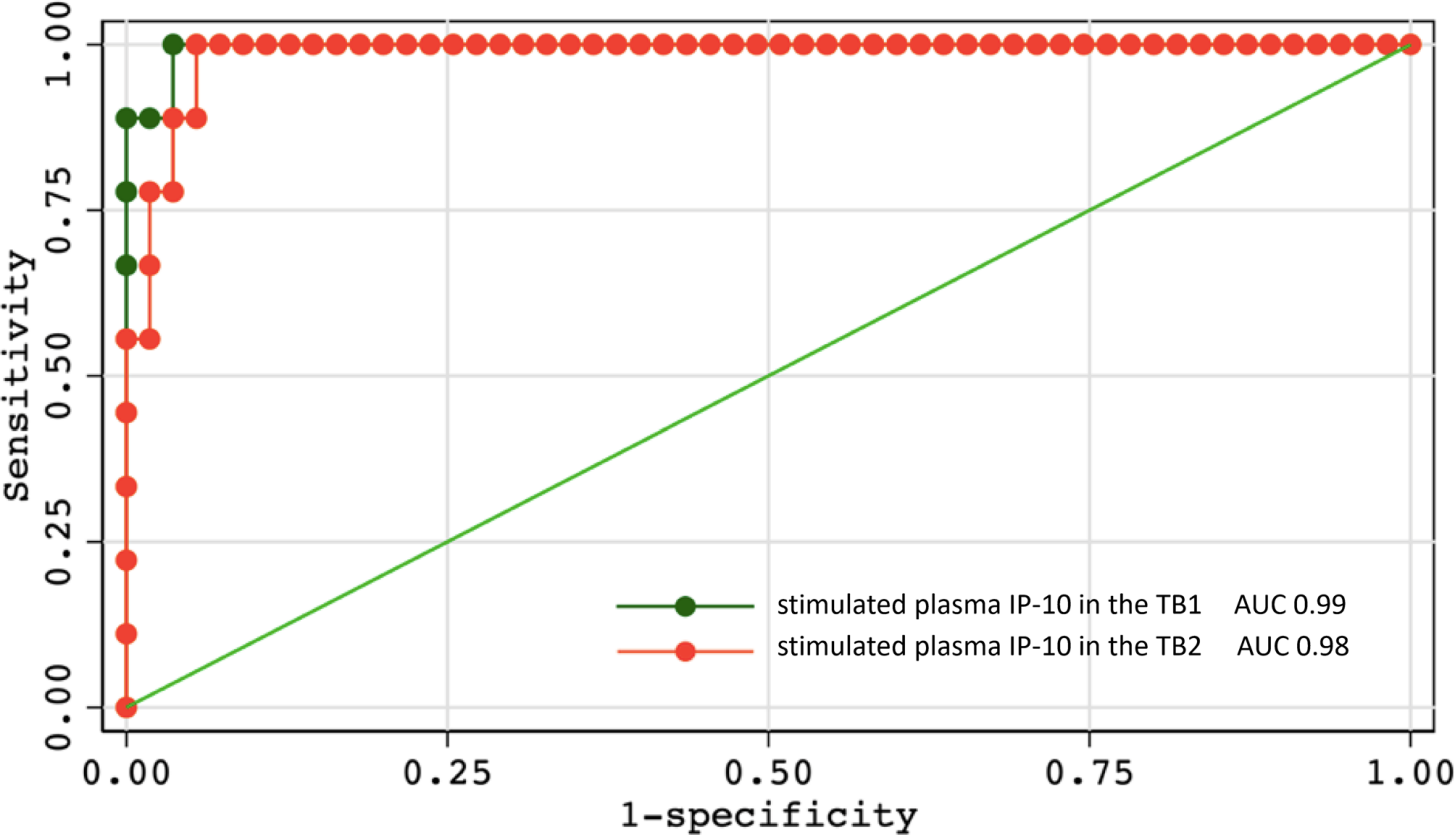
Receiver operating characteristic (ROC) analysis of stimulated plasma IP-10 in the TB1 and TB2 tubes for detecting latent TB infection. The area under the curve (AUC) was 0.99 (95% CI: 0.97–1.00; *p* < 0.001) for TB1 and 0.98 (95% CI: 0.93–1.00; *p* < 0.001) for TB2. IP-10, interferon-gamma inducible protein 10.

**Fig. 3:**
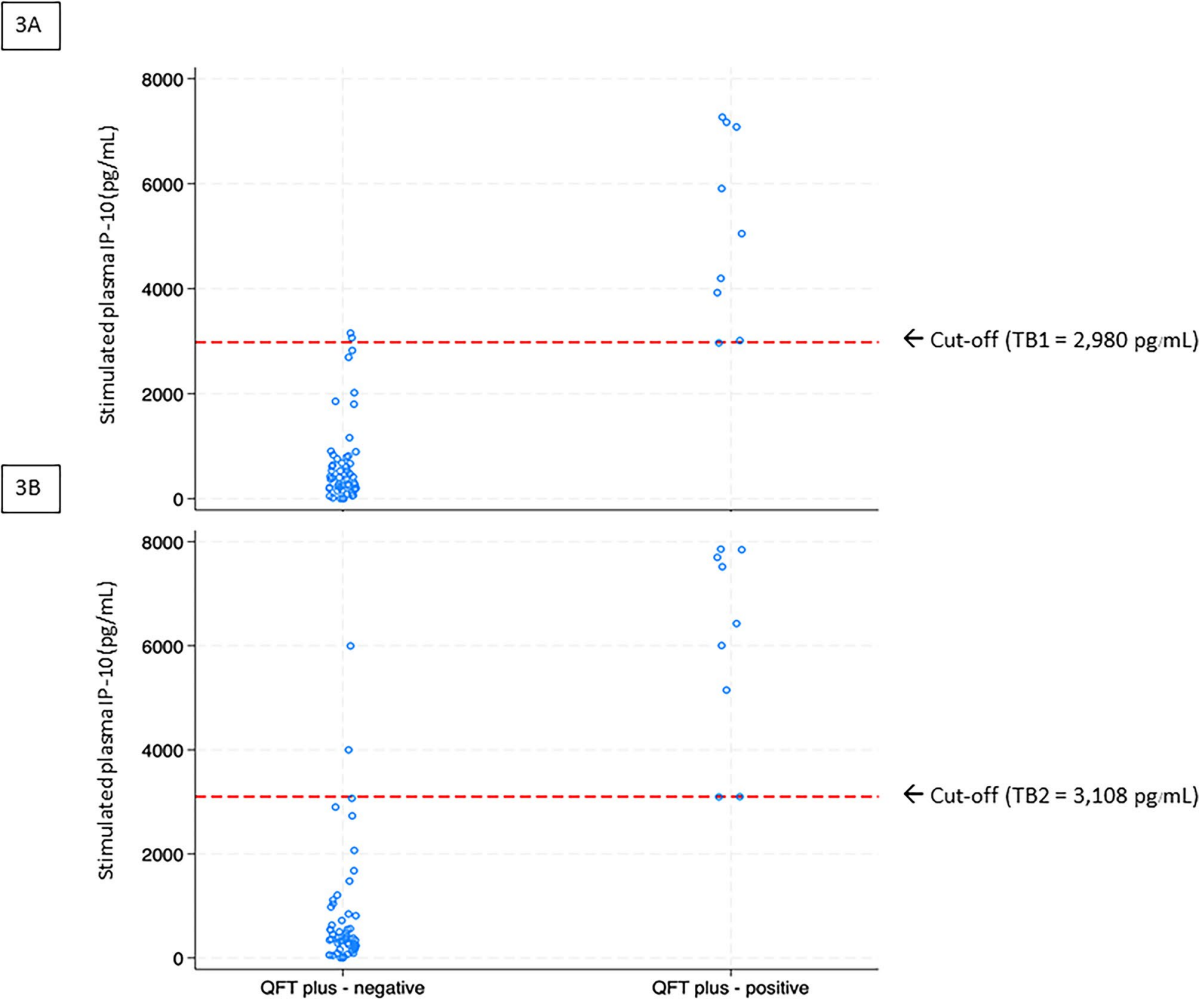
Scatter plots showing stimulated plasma IP-10 concentrations in QuantiFERON-TB Gold Plus–negative and QuantiFERON-TB Gold Plus–positive HCWs in TB1 (**A**) and TB2 (**B**) tubes. Each dot represents an individual participant. The horizontal dashed line indicates the optimal cut-off value for stimulated plasma IP-10 (2,980 pg/mL for the TB1 tube [Figure 3A] and 3,108 pg/mL for the TB2 tube [Figure 3B]). QFT-Plus, QuantiFERON-TB Gold Plus.

Stimulated plasma IP-10 showed excellent diagnostic performance, with area under the curves (AUCs) of 0.99 (95% CI: 0.97–1.00; *p* < 0.001) for TB1 and 0.98 (95% CI: 0.93–1.00; *p* < 0.001) for TB2 (Fig. 2). For TB1, the optimal cut-off value was ≥ 2,980.0 pg/mL, and classification based on this threshold is shown in Table 2; Fig. 3. At this cut-off, the test yielded a sensitivity of 100.0%, specificity of 96.0%, positive likelihood ratio (LR+) of 27.50 (95% CI: 7.10–107.00), diagnostic odds ratio (DOR) of 406.60 (95% CI: 18.90–8,756.40), and a diagnostic accuracy of 96.8%. For the TB2 tube, the optimal threshold for predicting LTBI was ≥ 3,108 pg/mL, with corresponding classification also shown in Table 2; Fig. 3. At this cut-off, plasma IP-10 demonstrated a sensitivity of 100.0%, specificity of 94.5%, LR + of 18.35 (95% CI: 6.10–55.00), DOR of 285.00 (95% CI: 13.60–5,960.00), and a diagnostic accuracy of 95.3%. Additional diagnostic indices for both tubes are summarized in Table 3.

**Table 2:** Diagnosis of LTBI using stimulated plasma IP-10, with QuantiFERON-TB Gold Plus (QFT-Plus) as the reference standard in 64 healthcare workers.

Stimulated plasma IP-10 cut-off	Gold standard QFT-Plus		Total HCWs
	Positive	Negative	
In TB1 tube			
≥ 2,980 pg/mL	9	2	11
< 2,980 pg/mL	0	53	53
In TB2 tube			
≥ 3,108 pg/mL	9	3	12
< 3,108 pg/mL	0	52	52

LTBI, latent tuberculosis infection; IP-10, interferon gamma inducible protein 10; QFT-Plus, QuantiFERON-TB Gold Plus; HCWs, health care workers.

**Table 3:** Diagnostic performance metrics for LTBI derived from stimulated plasma IP-10 in TB1 and TB2 tubes.

Stimulated plasma IP-10	Sensitivity (%)		Specificity (%)		Accuracy (%)		PPV (%)		NPV (%)
Cut-off in TB1 tube									
≥ 2,980 pg/mL	100.0		96.0		96.8		81.8		100.0
Cut-off in TB2 tube									
≥ 3,108 pg/mL	100.0		94.5		95.3		75.0		100.0
**LR and DOR with 95% CI**									
**Stimulated plasma IP-10**	**LR+**	**95% CI**		**LR-**		**95% CI**	**DOR**	**95% CI**	
Cut-off in TB1 tube									
≥ 2,980 pg/mL	27.50	7.10–107.00		0.05		0.00–0.78	406.60	18.90-8,756.40	
Cut-off in TB2 tube									
≥ 3,108 pg/mL	18.35	6.10–55.00		0.05		0.00–0.79	285.00	13.60-5,960.00	

LTBI, latent tuberculosis infection; PPV, positive predictive value; NPV, negative predictive value; LR+, positive likelihood ratio; LR-, negative likelihood ratio; DOR, diagnostic odd ratio; CI, confidence interval.

The average cost of stimulated plasma IP-10 testing was approximately 500 Thai baht per participant. This estimate was derived from the total cost of a commercial human IP-10 ELISA kit (Invitrogen™, Thermo Fisher Scientific, USA) and associated reagents, divided by the effective number of samples processed per kit, accounting for duplicate measurements and the inclusion of standard controls. In comparison, the cost of IGRAs was higher, at approximately 2,500 Thai baht per participant. Costs related to labor, equipment, overhead, and other indirect expenses were not included in this estimation.

Among the nine IGRA-positive HCWs, four (44.4%) initiated tuberculosis preventive therapy (TPT) according to the updated CDC-recommended regimens^34^, while five declined treatment. During follow-up, one of the five untreated individuals (20.0%) progressed to active pulmonary TB 16 months later, whereas no cases of active TB were observed among those who received TPT. In contrast, none of the 55 HCWs in the LTBI-negative group developed active TB during the 2-year follow-up period. Notably, stimulated plasma IP-10 demonstrated 100% sensitivity compared with IGRA; therefore, the classification of LTBI cases was identical between the two methods, and the longitudinal outcomes were consistent across both groups.

In additional analyses, HCWs with LTBI exhibited slightly higher, but non-significant, median concentrations of TB antigen–serum IP-10 compared with LTBI-negative HCWs (QFT-Plus TB1: 32.4 [15.2–61.5] vs. 22.2 [11.4–52.6] pg/mL; QFT-Plus TB2: 43.7 [18.3–70.8] vs. 34.0 [14.2–58.7] pg/mL; all *p* > 0.05). No significant correlations were observed between serum IP-10 levels and stimulated plasma IFN-γ concentrations in either the TB1 (*r* = 0.021, *p* > 0.05) or TB2 (*r* = 0.028, *p* > 0.05) tubes.

## Discussion

This study demonstrates the diagnostic value of an in vitro stimulated plasma IP-10 assay for detecting LTBI in HCWs. Antigen-stimulated IP-10 levels correlated strongly with IFN-γ and were significantly higher in LTBI-positive than LTBI-negative HCWs. The assay showed excellent discriminatory performance, with high AUC values. Using cut-offs of ≥ 2,980 pg/mL (TB1) or ≥ 3,108 pg/mL (TB2), stimulated plasma IP-10 achieved robust sensitivity, specificity, LR+, DOR, and overall accuracy. These results highlight stimulated plasma IP-10 as a promising and effective tool for LTBI screening, particularly in high-risk populations such as HCWs.

Currently, LTBI in healthcare personnel is primarily detected using TST and IGRAs. The TST is inexpensive, with sensitivity 71–94% and specificity 27–82.1%^17,20^, but requires two visits and may yield false negatives (booster phenomenon) or false positives in BCG-vaccinated or NTM-exposed individuals^13–15,18–20^. IGRAs, offering higher specificity (up to 96%) and faster results, are preferred but show suboptimal sensitivity (67–90%), especially in immunocompromised hosts, and demand well-equipped labs, complex procedures, trained personnel, and higher costs^13,14,16,17,20,35^. Consequently, IGRAs remain costly and are not widely implemented in low-income countries, including Thailand^17,23^.

To overcome TST and IGRA limitations, Rubbo et al. (2012) evaluated TB antigen–specific cytokines in 70 HCWs in France, a low TB–incidence country^30^. Cytokines measured in QFT cell-free supernatants—including monokine induced by IFN-γ, IL-2, IL-15, and IP-10—effectively distinguished LTBI-positive from LTBI-negative HCWs. Plasma IP-10, with higher expression and stability than IFN-γ, showed high diagnostic performance at a cut-off of 1,259 pg/mL, achieving 100% sensitivity and 85.7% specificity. It also reclassified 13 of 41 (31.7%) HCWs from the indeterminate group as LTBI-positive.

In this study, we evaluated stimulated plasma IP-10 from QFT-Plus supernatants for LTBI detection among HCWs in Thailand, using IGRAs as the reference standard^11,33^. Plasma IP-10 strongly correlated with IFN-γ (*r* = 0.719 for TB1, *r* = 0.768 for TB2; *p* < 0.0001; Fig. 1) and demonstrated excellent discrimination (Fig. 2). Using cut-offs of ≥ 2,980.0 pg/mL for TB1 and ≥ 3,108.0 pg/mL for TB2, IP-10 achieved 100% sensitivity, 96.0% and 94.5% specificity, LR + of 27.50 and 18.35, and overall accuracy of 96.8% and 95.3% (Table 3). Over 2 years, 20.0% of LTBI-positive individual (defined by either IGRA or stimulated plasma IP-10 positivity) who declined TPT developed active TB, whereas no LTBI-negative cases progressed.

Another noteworthy observation from our study is that the average laboratory cost per participant for measuring stimulated plasma IP-10 using an IGRA-based protocol was approximately fivefold lower than QFT-Plus assay. While this may superficially suggest that IP-10 testing could be more cost-effective than IGRA, it is important to note that our measurements relied on IGRA stimulation, and thus the observed cost advantage cannot be directly generalized to a standalone IP-10 assay. Nevertheless, several research-grade and near-commercial approaches are being explored to measure IP-10 directly without relying on IGRA, including whole blood stimulation with IP-10 ELISA, antigen-specific stimulation using ESAT-6/CFP-10, and multiplex cytokine assays^27,36,37^. These emerging platforms hold the potential to reduce the cost per participant for IP-10 testing in the future, supporting its feasibility as a practical alternative for LTBI screening in HCWs, particularly in high TB-burden, BCG-vaccinated settings.

In our study, non-stimulated serum IP-10 concentrations did not significantly correlate with stimulated plasma IGRA levels, as average serum IP-10 levels among HCWs were too low to be reliably detected. This may be explained by the direct measurement of IP-10 in serum, which does not provide sufficient lymphocyte stimulation. Therefore, serum is not recommended as a suitable platform for IP-10–based assays in the diagnosis of LTBI, a finding consistent with recently published data by Druszczynska et al.^38^.

When comparing the diagnostic performance of stimulated plasma IP-10 with the TST and IGRAs, the literature indicates that IP-10 yields fewer false-positive results in individuals who have received BCG vaccination or been exposed to NTM^13–15,18,19,23,27–29^. Compared with IGRAs, IP-10 testing offers several advantages. First, IP-10 is produced at higher levels than IFN-γ, resulting in stronger signal detection, enhanced assay sensitivity, and improved reproducibility^24,25^. Second, IP-10 is more stable in plasma supernatants, reducing laboratory complexity and minimizing variability from sample handling. Third, IP-10 testing may represents a relatively low-cost alternative in terms of assay-related expenses^26,30^, however, in the present study, IP-10 measurement was performed using IGRA-based stimulation, which limits direct cost comparisons. Lastly, rapid formats—such as lateral flow platforms or dried plasma spots—enable simpler, faster, and potentially more cost-efficient workflows, particularly in large-scale studies or in populations with low IFN-γ responses, including children and immunocompromised individuals. Collectively, these attributes highlight the utility of IP-10 as a practical and scalable biomarker for LTBI screening^39,40^.

**Fig. 4:**
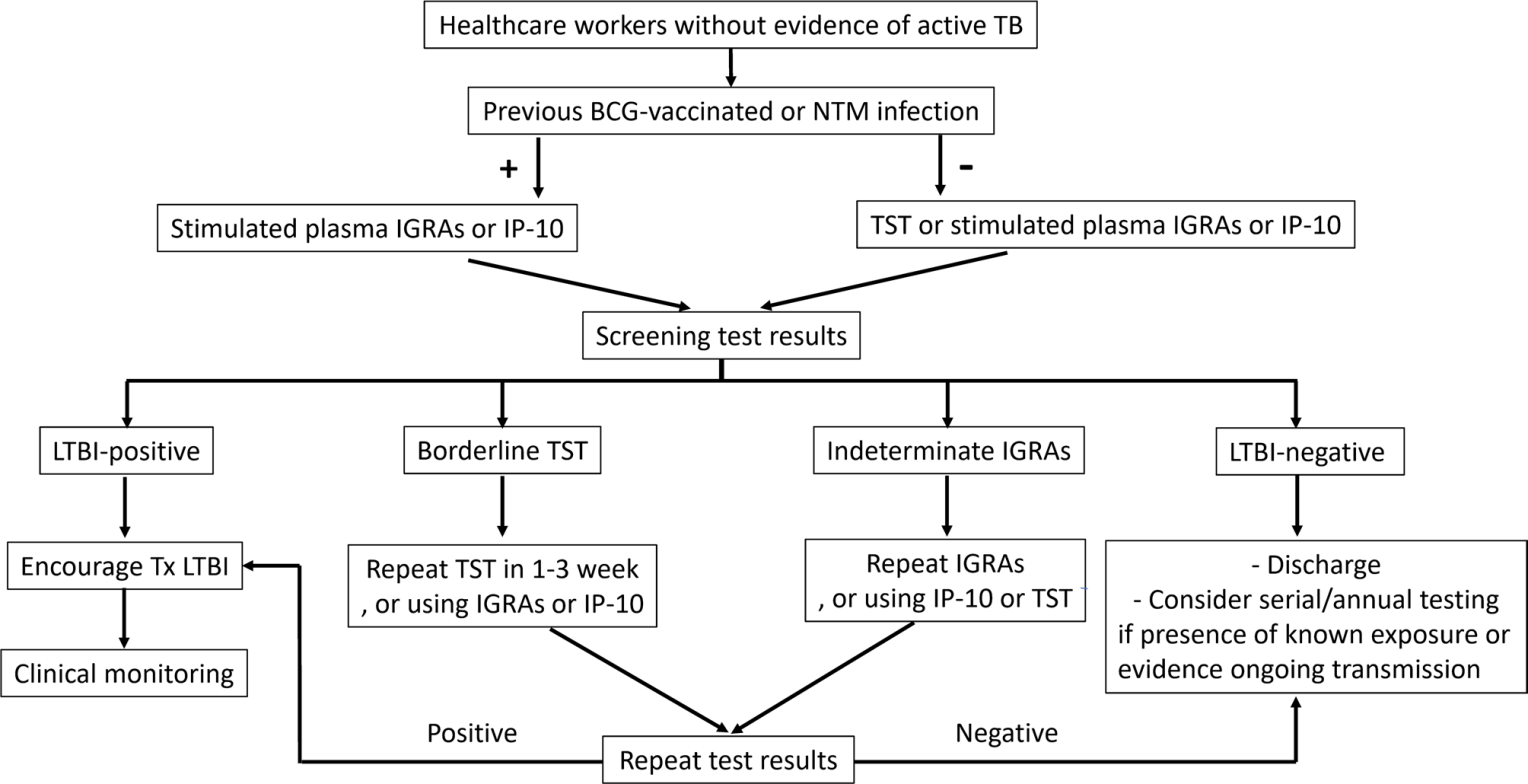
Proposed algorithm for the diagnosis and management of LTBI in healthcare workers. TB, tuberculosis; BCG, Bacillus Calmette–Guérin; NTM, non-tuberculous mycobacteria; IGRAs, interferon-gamma release assays; IP-10, interferon-gamma inducible protein 10; TST, tuberculin skin test; LTBI, latent tuberculosis infection.

Based on the benefits and limitations of the three LTBI diagnostic tests reported in the literature, together with evidence from this study, we propose an algorithm for the diagnosis and management of LTBI in HCWs, as illustrated in Fig. 4.

To our knowledge, this is the first study to comprehensively evaluate and demonstrate the diagnostic performance of stimulated plasma IP-10 assays for detecting LTBI among HCWs in a high–tuberculosis-incidence country with universal BCG vaccination. Major strengths include: (1) the assessment of the correlation between both stimulated plasma and non-stimulated serum IP-10 with IGRA concentrations; (2) the determination of sensitivity, specificity, and additional diagnostic indices, including LRs, DORs, and overall accuracy at optimal IP-10 cut-off values, with the study findings regarding the benefits of IP-10 aligning with the existing literature in populations beyond HCWs; and (3) the illustration of two-year clinical follow-up outcomes among LTBI-positive and LTBI-negative HCWs, supporting the diagnostic performance and reliability of stimulated plasma IP-10.

However, several limitations of this study should be acknowledged. First, this is a single-center study with a relatively small number of LTBI-positive HCWs, which may have contributed to wide 95% confidence intervals for LRs, DORs, and accuracy estimates. Second, the absence of indeterminate IGRA results prevented assessment of the potential reclassification performance of IP-10. This may reflect differences in the definition of indeterminate results compared with previous studies^30^, as well as the inclusion of only immunocompetent HCWs and the use of an updated IGRA version^41^. Third, limitations inherent to IP-10 measurement should be noted, including: (1) variability in measurement platforms, such as multiplex assays and both commercial and in-house ELISA kits; (2) differences in sample dilution, which may not always align with optimal standard curve ranges, leading to variable cut-off values across studies; and (3) dependence on QFT-Plus supernatants, meaning the assay currently relies on IGRA stimulation (IGRA-based IP-10 assay)^25,26,30^. Fourth, we acknowledge that using QFT-Plus as the reference standard represents an imperfect surrogate for LTBI, and fifth, we lacked external validation data to confirm the accuracy of the proposed IP-10 cut-off values. Finally, the cost-effectiveness of IP-10 testing has not yet been formally evaluated. Further studies with a larger sample size, a multicenter design, and formal cost-effectiveness analyses are warranted to validate, confirm and support these findings.

## Conclusions

Stimulated plasma IP-10 assays show high diagnostic performance for detecting LTBI in HCWs and represent a simpler alternative to IGRAs. Thus, IP-10 may be a valuable LTBI screening tool, especially in high TB-burden settings with universal BCG vaccination.

## Materials and methods

### Study design and participants

This longitudinal cohort study with baseline testing was conducted at Songklanagarind Hospital, Thailand, from December 2021 to October 2024. Healthy, HIV-negative HCWs aged ≥ 18 years with direct contact with TB patients were enrolled. Exclusion criteria included symptoms or chest X-ray findings suggestive of active TB, prior TB diagnosis or treatment, and immunocompromised conditions (e.g., immunosuppressive therapy, malignancy, or radiotherapy).

The study was approved by the Ethics Committee of the Faculty of Medicine, Prince of Songkla University (REC 64-363-14-1), adhered to the Declaration of Helsinki and Good Clinical Practice. This trial was registered in the Thai Clinical Trials Registry (TCTR20251229008) on December 29, 2025, with retrospectively registered. All participants provided written informed consent, and their medical history, physical examination, HIV status, and chest X-ray results were recorded.

## Methods

### Blood collection and processing

A total of 6 mL of peripheral blood was collected from each participant (2 mL for serum, 4 mL in heparinized tubes for IGRA). Serum was separated by centrifugation (1500 × g, 20 min) and stored at − 20 °C for IP-10 analysis. Heparinized blood was processed within 4 h using QuantiFERON-TB Gold Plus (QFT-Plus; Qiagen, Hilden, Germany), and aliquoted into four 1 mL tubes: Nil (negative control), TB1 (CD4 + stimulation), TB2 (CD4 + and CD8 + stimulation), and mitogen (positive control). Tubes were incubated at 37 °C for 16 h, centrifuged (3000 × g, 15 min), and plasma stored at − 20 °C for IFN-γ and IP-10 ELISA^10,39,42,43^.

### IFN-γ level analysis from stimulated plasma

For IFN-γ measurement according to the QFT-Plus manufacturer’s instructions, briefly, 50 µL of diluted anti-human IFN-γ-HRP antibody and 50 µL of standards or test plasma (Nil, TB1, TB2, Mitogen) were added to each well in duplicate. Plates were shaken, incubated in the dark for 2 h, washed, incubated with substrate for 30 min, stopped, and read at 450 nm. Concentrations were calculated in pg/mL using QFT-Plus software (40 pg/mL = 1 IU/mL)^39^. LTBI was defined as TB1 or TB2 ≥ 0.35 IU/mL and ≥ 25% above Nil without active TB; uninfected if responses < 0.35 IU/mL or < 25% above Nil. Indeterminate results were defined as mitogen minus Nil < 0.50 IU/mL or Nil > 8.0 IU/mL. Participants with positive IGRA were advised to receive LTBI treatment and follow-up^10,41,42,44^.

### IP-10 level analysis from stimulated plasma and serum

IP-10 levels were measured in serum and stimulated plasma (QFT-Plus supernatants) using a human IP-10 ELISA kit (Invitrogen™, Thermo Fisher Scientific, USA). Each kit contains sufficient reagents to process multiple participant samples, with all measurements performed in duplicate and including standard controls, allowing efficient use of the kit for a larger number of samples.

The assay employs a solid-phase sandwich ELISA with microtiter wells pre-coated with a monoclonal anti–IP-10 capture antibody. A standard curve was prepared from serially diluted IP-10 standards ranging 500–7.8 pg/mL, with Standard Diluent Buffer as the blank. Stimulated plasma supernatants from TB1 and TB2 tubes were diluted 1:10 in Standard Diluent Buffer. Fifty microliters of standards, controls, and diluted samples were added per well, followed by 50 µL of biotinylated anti-IP-10 detection antibody. The plates were incubated for 3 h at room temperature to allow IP-10 to bind both the immobilized capture antibody and the biotinylated detection antibody. After incubation, the wells were washed four times to remove unbound material. Subsequently, 100 µL of Streptavidin-HRP working solution was added to each well, and the plates were incubated for 30 min at room temperature, enabling the HRP-conjugated streptavidin to bind to the biotinylated antibody and complete the sandwich complex. Following another four wash cycles, 50 µL of Stabilized Chromogen substrate was added. The plates were incubated for 30 min in the dark to allow colour development, which is proportional to the amount of IP-10 present. The reaction was then stopped by adding 100 µL of Stop Solution, resulting in a yellow end-point colour. Absorbance was measured at 450 nm within 2 h using a microplate reader. IP-10 concentrations were determined from the optical density values using a log–log standard curve^38^.

Participants were clinically followed up for a period of 24 months after enrollment, with scheduled assessments at 6, 12, 18, and 24 months to monitor for the development of active TB. Follow-up included review of medical records, symptom assessment, and diagnostic testing if clinically indicated.

### Statistical analysis

The sample size was calculated using an accuracy-based formula, assuming 35% LTBI prevalence among Thai HCWs^7^ and 90% accuracy, yielding 60 participants. Accounting for 10% potential dropout, 66 participants were targeted. Continuous variables were reported as mean ± SD and categorical as frequency (%). Student’s t-test, chi-square, or Fisher’s exact test were used for comparisons. Pearson’s correlation assessed associations between IGRA and IP-10 (TB1/TB2) levels, visualized with scatterplots. Using IGRA as reference, stimulated plasma IP-10 diagnostic performance was evaluated via ROC curves, AUC, and optimal cut-offs; sensitivity, specificity, predictive values, likelihood ratios (LRs), diagnostic odd ration (DOR), and accuracy were calculated. Analyses were performed in Stata 17.0, with *p* < 0.05 considered significant.

## Data Availability

The data produced and analyzed during in the present study can be obtained from the corresponding author upon request, subject to reasonable conditions.
